# Torpor patterns in common hamsters with and without access to food stores

**DOI:** 10.1007/s00360-017-1093-6

**Published:** 2017-04-17

**Authors:** Carina Siutz, Eva Millesi

**Affiliations:** Department of Behavioural Biology, University of Vienna, Althanstrasse 14, 1090 Vienna, Austria

**Keywords:** Hibernation, Body temperature, Food stores, Short torpor bout, Common hamster

## Abstract

Hibernating species significantly reduce energy expenditure during winter by entering torpor. Nevertheless, the various benefits of hibernation might be counteracted by negative effects of torpor such as immune depression, oxidative stress, or neuronal impairment. Considering these trade-offs, adequate energy reserves could allow animals to reduce the time spent in torpor or the extent of metabolic depression. Common hamsters use food stores during hibernation and previously documented high individual variations in body temperature patterns during winter could, therefore, be related to differences in external energy reserves. In this study, we manipulated the availability of food stores under laboratory conditions to investigate potential effects on hibernation patterns. Female hamsters were kept in artificial burrows in climate chambers and subcutaneous temperature was recorded using implanted data loggers. One group had access to large food stores, whereas another group received daily food portions which were removed on the next day if not consumed. Almost all hamsters without access to food stores hibernated, while less than half of the individuals with food stores entered deep torpor. Individuals without food hoards additionally expressed more short torpor bouts and exhibited lower minimum subcutaneous temperatures during torpor than those with food stores. Thus, individuals confronted with lacking food reserves were more likely to hibernate and additionally saved energy by entering short torpor bouts more frequently and remaining at lower subcutaneous temperature both during torpor and euthermic periods. In conclusion, our results demonstrate that food store availability affects torpor expression and also highlight variation in torpor patterns and energy-saving strategies in common hamsters.

## Introduction

Hibernation is a highly efficient way to save energy during periods of low ambient temperatures and food shortage due to metabolic depression and reduced body temperature (*T*_b_) during multiday torpor bouts (Geiser 2004; Heldmaier et al. 2004; Heldmaier and Ruf 1992; Ruf and Geiser 2015; Ruf and Heldmaier 1992). To meet energetic demands during hibernation, individuals can use body fat reserves as energy supply (Dark 2005; Florant and Healy 2012; Humphries et al. 2003b; Sheriff et al. 2013) or rely on food stores as external energy reserves (French 2000; Humphries et al. 2003b; Munro et al. 2008). In addition to highly reduced energy expenditure through torpor expression (e.g., Heldmaier and Ruf 1992; Levesque and Tattersall 2010; Ruf and Geiser 2015; Ruf and Heldmaier 1992), other benefits like reduced water loss and parasite load have been documented (Geiser and Brigham 2012). Furthermore, being able to stay in a hibernaculum for several months could minimize predation risk as indicated by higher survival rates during winter compared to the active season (Bieber et al. 2012; Bryant and Page 2005; Geiser and Turbill 2009; Lebl et al. 2011; Turbill et al. 2011). Accordingly, in some hibernating species, individuals in good condition, indicating high internal energy reserves, were found to express more frequent, longer, and/or deeper torpor bouts (Hallam and Mzilikazi 2011; Kobbe et al. 2011; Stawski and Geiser 2010; Vuarin et al. 2013).

Despite these evident advantages, negative effects of deep torpor such as, e.g., immune depression (Franco et al. 2013; Prendergast et al. 2002), oxidative stress (Carey et al. 2000; Giroud et al. 2009), ischemia (Carey et al. 2003), reduced synaptic efficacy (Strijkstra et al. 2003), or impaired memory retention (Millesi et al. 2001) have also been suggested. Hence, trade-offs between benefits and potential costs of torpor could result in adjusting torpor expression according to available energy reserves. In some fat-storing hibernators, individuals with higher body mass prior to hibernation showed longer euthermic periods and higher *T*_b_ during torpor (Bieber et al. 2014; Zervanos et al. 2013). Similarly, among food-storing hibernators individuals responded to food supplementation and high food availability with reduced duration and/or depth of torpor expression (French 2000; Humphries et al. 2003a; Landry-Cuerrier et al. 2008; Munro et al. 2005). These results indicate that hibernators can reduce potential costs of torpor by hibernating for shorter periods and/or less intensely when sufficient energy reserves are available.

Common hamsters (*Cricetus cricetus*) are facultative hibernators and use food stores during winter which are accumulated during the active season (Eibl-Eibesfeldt 1953; Niethammer 1982). Previous studies demonstrated a high individual variation in hibernation patterns under semi-natural and laboratory conditions (Wassmer 2004; Wassmer and Wollnik 1997; Wollnik and Schmidt 1995). In some individuals, *T*_b_ patterns during winter resembled those of obligate hibernators. Other individuals, however, showed extended euthermic periods followed by a short hibernation period or even remained continuously euthermic. In addition, we recently documented a high variation in the onset and duration of hibernation in free-ranging common hamsters (Siutz et al. 2016). Furthermore, females hibernated for shorter periods and spent less time in torpor than males. This sex difference could be related to the availability of external energy reserves, because food caching activities are much more pronounced in females compared to males, indicating larger food stores in females’ hibernacula (Siutz et al. 2012, 2016). In field studies, however, information about the quantity and quality of stored food is lacking.

In the present study, we aimed at investigating whether access to food stores in the hibernaculum affects torpor expression by manipulating food hoard availability under laboratory conditions in female common hamsters. We compared individuals that could cache large amounts of food sufficient to survive until spring to others that received food on a daily basis and were prevented from accumulating stores by removing food remains the following day. If hamsters adjust torpor expression in relation to external energy reserves, we would expect that the individuals with access to food stores would spend less time in torpor compared to hamsters without reliable food availability.

## Materials and methods

### Animals and housing conditions

Twenty-four female common hamsters (aged 9 months, partly siblings but max. 2 individuals from the same litter), obtained from a laboratory breeding colony in Strasbourg, France (Chronobiotron UMS 3415, Centre de Neurochimie), were individually housed in transparent plastic cages (99 × 51.5 × 36 cm; Ferplast, Maxi Duna Multy) equipped with an artificial burrow system consisting of three chambers (one used as nest box, one to store food, and another used for defecation; each 23 × 16 × 14 cm) that were connected via plastic tubes. The boxes were equipped with accessible lids. The animals were kept at 19 ± 1 °C under natural photoperiod prior to the experiment and received food pellets (rodent standard pellets, Ssniff V2233, 3% fat, 17% protein, 13% fibre, 16.6 MJ/kg gross energy content; Ssniff Spezialdiäten GmbH, Soest, Germany) and water ad libitum. Starting 3 weeks prior to the experiment onset (23rd December 2013), we gradually reduced ambient temperature to 6 °C to allow the animals an acclimatisation period. Photoperiod length corresponded to natural conditions, i.e., we followed the natural decrease in photoperiod. This initial phase resembled natural conditions as burrow temperatures in free-ranging hamsters usually do not drop below 10 °C before December (Siutz et al., unpublished data). Throughout the experiment, the hamsters were exposed to cold ambient temperature (6 ± 0.5 °C) and short photoperiod (6L:18D, lights on at 0800 h). Starting in late March, photoperiod was steadily increased to approximate natural photoperiod again. When hamsters were not in torpor, they usually showed activity outside their burrows. We could, therefore, detect if an animal was active by placing a small piece of cotton wool at the burrow entrance, as it was moved whenever an individual was outside the burrow. This was recorded on a daily basis.

### Experimental design

The animals were assigned to 2 groups of 12 individuals and body mass did not differ between the groups prior to the experimental period (store: 208 ± 11 g, *n* = 9; daily food: 218 ± 8 g, *n* = 12; *p* = 0.451). Sibling pairs were not in the same group. One group received 2000 g pellets (Ssniff V2233) to hoard at experimental onset. This amount is known to be sufficient to endure the experimental period without the use of torpor (Siutz et al., unpublished data), and is supported by the fact that none of the individuals completely consumed the provided food. The hamsters could cache the pellets and store them inside the food chamber. The other group had no access to food stores but received 20 g pellets (Ssniff V2233) per day. Remaining food was removed each day and the food chamber was checked daily by lifting the lid to ensure that no pellets were stored. The food chamber of the group with hoards was checked at the same intervals but without removing the food, to control for potential effects of opening the lid. The other two chambers were checked at regular intervals in both groups (approx. weekly; when an individual was active as detected by an absent cotton wool). None of the animals expressed torpor before the onset of the experiment.

The end of the experiment (9th April 2014) was set 2 weeks after the last torpor bout was observed (to ensure that all individuals terminated hibernation) and resembled the date when most hamsters at our field site had resumed above-ground activity (Siutz et al. 2016). The duration of the experimental period was within the range of hibernation periods of free-ranging common hamsters as particularly females are known to enter hibernation between late December and early January (Siutz et al. 2016).

Two individuals of the group with food stores had to be excluded from the study due to veterinary treatments and another one due to recording failure of subcutaneous temperature (*T*_sub_). This resulted in a sample size of nine individuals in this group.

### Hibernation patterns

We recorded *T*_sub_ at 90-min intervals using temperature data loggers (iButtons, DS1922L-F5#, range −40 to + 85 °C, accuracy: ±0.5 °C, Maxim Integrated Products International, Dublin, Ireland). About 2 months prior to the experimental onset (17th October 2013), the hamsters were transported (~15 min) to a veterinary clinic where the iButtons (coated in Elvax ethylene vinyl acetate resins, DuPont, and paraffin; gas-sterilised; potted mass: ~4.5 g) were implanted subcutaneously in the neck region (dorsal, between the scapulae) under isoflurane anaesthesia. This method has proved successful in this species (Siutz et al. 2016). After the experiment, the iButtons were removed using the same technique. Since the measured subcutaneous temperature in our study might slightly deviate from core temperature, we used *T*_sub_ when referring to our results and *T*_b_ when referring to other studies.

**Fig. 1:**
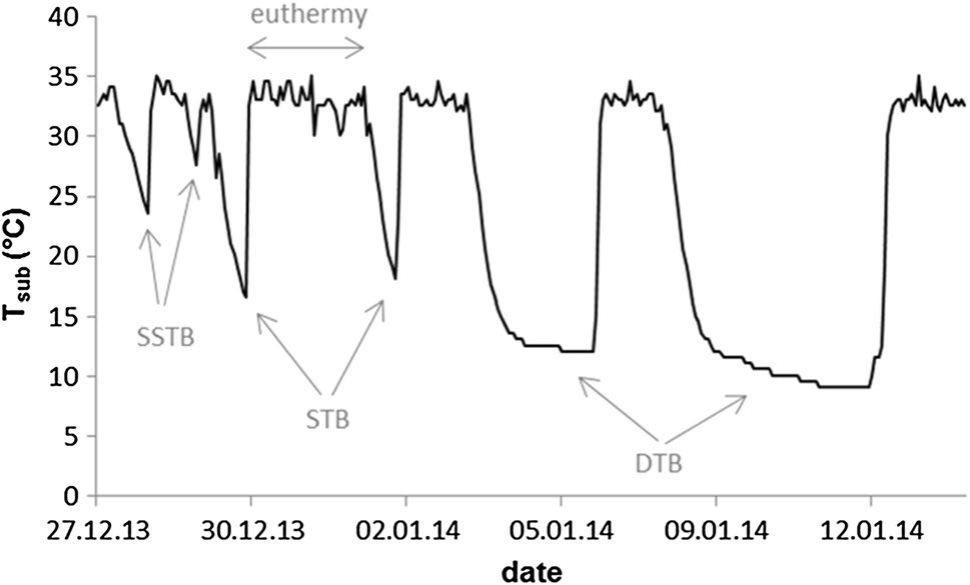
Subcutaneous temperature (*T*_sub_) of a common hamster under laboratory conditions demonstrating the three types of torpor bouts: deep torpor bouts (DTB), short torpor bouts (STB), and short and shallow torpor bouts (SSTB) alternating with euthermic periods

Torpor was defined as the interval between the first decrease in *T*_sub_ below 30 °C and the first increase of *T*_sub_ above 30 °C. Deep torpor bouts (DTBs) were characterized by *T*_sub_ below 20 °C (mean ± SE: 9.3 ± 0.1 °C, *n* = 15) and a duration exceeding 24 h (3.6 ± 0.2 days). In addition, two other, clearly distinguishable, types of torpor (Fig. 1) could be identified and were defined according to Wassmer and Wollnik (1997) as short torpor bouts (STBs) with *T*_sub_ drops below 20 °C (17.7 ± 0.4 °C, *n* = 16) but a duration shorter than 24 h (13.7 ± 0.6 h), and short and shallow torpor bouts (SSTBs) in which *T*_sub_ remained above 20 °C (27.3 ± 0.2 °C, *n* = 21) for a few hours (4.9 ± 0.2 h).

For each torpor type, we analysed the following parameters: number, total duration of torpor bouts (calculated in hours, expressed as days), bout duration (calculated in hours, expressed as days), minimum *T*_sub_ (lowest value of *T*_sub_ during torpor bouts), and mean *T*_sub_ (beginning from the sampling interval when *T*_sub_ decreased below 30 °C until it had reached 30 °C again). In addition, we calculated the absolute minimum *T*_sub_ (an individual’s lowest *T*_sub_ throughout the experimental period) and the mean *T*_sub_ during euthermy throughout the experimental period (*T*_sub_ above 32.5 °C calculated from the first to the last day of the experiment; this *T*_sub_ set point should ensure that neither the onset nor end of a torpor bout was included). Among individuals that hibernated, we analysed the duration of the hibernation period (days from the onset of the first to the termination of the last DTB).

### Statistics

Statistical analyses were conducted in R (R Development Core Team 2009). We initially performed linear models for the parameters number and total duration of torpor bouts, and hibernation duration and included experimental group (store versus daily food), and body mass prior to the experiment as well as their interaction as predictor variables. Since body mass had no effect on the respective response variable, it was removed from all models as this contributed to AIC (Akaike’s information criterion) reduction, and we performed Student’s *t* tests for group comparisons. Data distributions were tested for normality by Shapiro–Wilk tests. Not normally distributed parameters were transformed by taking the second root. Parameters that could not be transformed to achieve normal distribution were compared between groups by applying Mann–Whitney *U* tests. To analyse if the number of individuals entering torpor differed between groups we used Pearsons’s Chi^2^ test of counts. We computed linear mixed models (LMEs) for the parameters torpor bout duration, minimum *T*_sub_, and mean *T*_sub_. We included the date of each torpor bout onset to correct for potential seasonal effects and the experimental group as fixed effects, and individual identity as random effect to correct for repeated measurements. Model residuals were tested for normality using Shapiro–Wilk tests and for homoscedasticity using Levene tests. *p* values were obtained from ANOVA (Type III) tables. Significance level was set at *p* ≤ 0.05. Results are presented as means ± SE.

## Results

Almost all individuals (11 out of 12) that received daily food portions expressed deep torpor bouts (DTB), while only about half of the individuals (4 out of 9) with access to food stores entered deep torpor (*p* = 0.059; 91.7 versus 44.4% for the daily food and store group, respectively). Similarly, all but one hamster (91.7%, *n* = 11) without food stores expressed short torpor bouts (STBs) compared to 55.6% (*n* = 5) of the individuals with access to food stores (*p* = 0.160). All individuals showed short and shallow torpor bouts (SSTB) (Table 1). The number and total duration of DTBs did not correlate with the number or total duration of STBs and SSTBs, respectively (*p* > 0.105 in all cases). Among individuals that showed DTBs, none of the parameters differed significantly between the groups (Table 1). Likewise, hibernation duration was similar in both groups (store: 46 ± 7.1 days, daily: 35.8 ± 8.5 days; *p* = 0.379).

**Table 1:** Comparison of number, duration, and subcutaneous temperature (*T*_sub_) of deep torpor bouts (DTB), short torpor bouts (STB), and short and shallow torpor bouts (SSTB) between the two groups

Torpor type	Parameters	Group		*p* value
		Food stores	Daily portions	
DTB	*n*	4	11	
	Number	5.8 ± 2.3	5 ± 1.4	0.68
	Total duration (d)	20.8 ± 9.7	20.2 ± 6.2	0.907
	Mean duration (d)	3.2 ± 0.5	3.7 ± 0.2	0.188
	Minimum *T*_sub_ (°C)	9 ± 0.1	8.7 ± 0.2	0.348
	Mean *T*_sub_ (°C)	12.8 ± 0.9	11.8 ± 0.3	0.151
STB	*n*	5	11	
	Number	1.8 ± 0.6	2.5 ± 0.6	0.05
	Total duration (d)	1 ± 0.3	1.4 ± 0.3	0.05
	Mean duration (d)	0.6 ± 0.04	0.6 ± 0.03	0.578
	Minimum *T*_sub_ (°C)	17.4 ± 0.5	16.6 ± 0.6	0.553
	Mean *T*_sub_ (°C)	23.8 ± 0.3	22.8 ± 0.2	0.023
SSTB	*n*	9	12	
	Number	17.2 ± 3.9	36.1 ± 9.2	0.154
	Total duration (d)	3.4 ± 0.6	6.6 ± 1.6	0.132
	Mean duration (d)	0.2 ± 0.01	0.2 ± 0.01	0.315
	Minimum *T*_sub_ (°C)	23.4 ± 0.4	23.1 ± 0.4	0.652
	Mean *T*_sub_ (°C)	28.2 ± 0.1	28.1 ± 0.1	0.779

Values represent means ± SEM

Individuals without access to food stores, however, showed significantly more STBs, spent more time in STBs, and had lower mean *T*_sub_ during STBs compared to hamsters with food stores (Table 1). Mean bout duration and the minimum *T*_sub_ during STBs did not differ between the groups. Number, duration, and minimum temperature during SSTBs were also similar in both groups (Table 1). In addition, we compared torpor bouts lasting less than 24h (i.e., we combined STBs and SSTBs) between the groups. Neither bout number (store: 18.2 ± 3.8, daily: 38.4 ± 9.4; *p* = 0.121) nor total duration (store: 4 ± 0.6 d, daily: 7.9 ± 1.7 d; *p* = 0.075) differed between the groups.

**Fig. 2:**
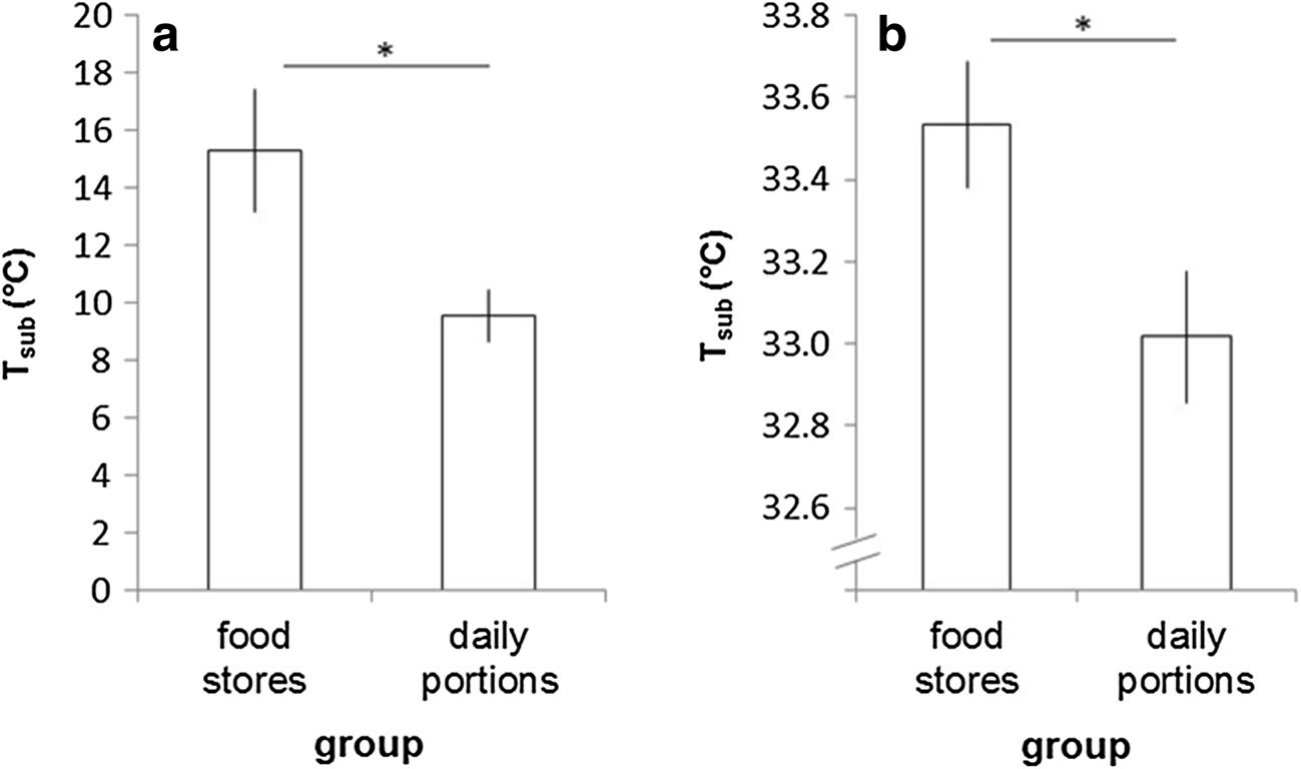
Minimum subcutaneous temperature (*T*_sub_) during torpor (**a**) and mean *T*_sub_ during euthermy (**b**) in individuals with and without access to food stores. Mean ± SE, *p ≤ 0.05

Minimum *T*_sub_ during torpor was significantly lower in individuals without access to food stores compared to those with external food reserves (*p* = 0.013, Fig. 2a). Furthermore, mean *T*_sub_ during euthermy differed significantly between the groups, showing lower temperatures in the group without food stores (*p* = 0.034, Fig. 2b).

**Fig. 3:**
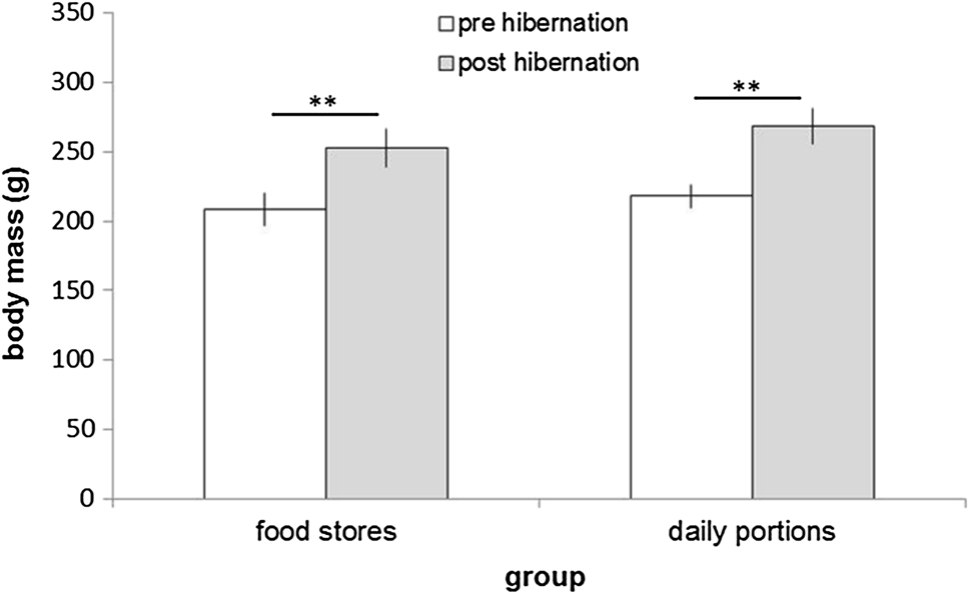
Body mass at the onset (pre-hibernation) and end (post-hibernation) of the experiment. Mean ± SE, **p ≤ 0.01

Body mass did not differ between the groups at the end of the experiment (*p* = 0.402; Fig. 3). All individuals gained body mass during the experimental period (store: 21.6 ± 3%, *p* = 0.009; daily food: 23.2 ± 3%, *p* = 0.003) and the relative mass increase was similar in both groups (*p* = 0.737).

## Discussion

By experimentally manipulating food store availability, we could show that almost all hamsters without food stores hibernated, indicating that unpredictable food availability might promote the use of torpor. Individuals with access to food stores and hence, permanent food availability, however, were less likely to hibernate as more than half of these animals did not enter deep torpor. Body mass prior to the experimental onset was similar between the groups and within the range of free-ranging individuals (Pluch et al. 2013). Variations in body mass among individuals in each group had no effect on torpor expression (as revealed by initial statistical analyses), so it is unlikely that differences in body condition affected hibernation performance. It seems, therefore, that in common hamsters, external energy reserves are essential in adjusting torpor expression and large amounts of food stores can allow individuals to avoid using torpor.

Effects of energy reserves on hibernation performance might be more pronounced in food-storing compared to fat-storing hibernators due to their greater energy-storing capacity (Humphries et al. 2003b), and several studies demonstrated that when food was abundant, torpor expression was reduced both in daily heterotherms and hibernators (reviewed in Vuarin and Henry 2014). In our study, however, neither the number or duration of deep torpor bouts (DTBs) nor *T*_sub_ during DTBs differed between the hibernating individuals of both groups. These results are in contrast to studies on other food-storing species like eastern chipmunks where individuals with large food stores spent less time in torpor, expressed shorter torpor bouts, and/or at higher *T*_b_ (French 2000; Humphries et al. 2003a; Landry-Cuerrier et al. 2008; Munro et al. 2005). One possible explanation for this difference might relate to the high variation in *T*_sub_ patterns expressed by common hamsters. In addition to DTBs, the hamsters in our study showed short (STB) and shallow (SSTB) torpor bouts. These torpor types also represent reduced energy expenditure, albeit not to the same extent as deep torpor due to higher metabolic rates. We found no relationships between the expression of DTBs and STBs or SSTBs, indicating that the individuals did not replace DTBs by increasing the number or duration of STBs or SSTBs. Temperature patterns in individuals that had hibernated represented a combination of all torpor types rather than alternative strategies (i.e., either DTBs or STBs/SSTBs). Therefore, our results demonstrate a high flexibility in torpor expression in common hamsters. Such flexibility has been suggested to be particularly adaptive when environmental conditions are unpredictable (Canale and Henry 2010), and although the underlying mechanisms are largely unknown, the availability of reliable energy reserves seems to play a major role in determining overwintering strategies.

Similar to DTBs, almost all hamsters without food stores showed STBs (the one that did not, though, showed DTBs). They expressed STBs more frequently, had lower mean *T*_sub_ during STBs, and also lower minimum *T*_sub_ during winter compared to individuals with food stores. These results indicate that hamsters without access to food stores expressed torpor more effectively. In addition, these individuals even showed lower *T*_sub_ during euthermy. Subcutaneous temperature could be affected by insulation in the nest box. We did not record the duration of activity outside the burrows, but as most of the individuals without food stores hibernated, it is very unlikely that they spent more time outside the nest box than the others. Lower euthermic *T*_b_ during winter compared to summer was shown in eastern chipmunks and could allow individuals to slightly reduce energy expenditure (Levesque and Tattersall 2010). Drops in normothermic *T*_b_ prior to the first deep torpor bout have been shown in both daily heterotherms (Christian and Geiser 2007) and hibernators (Kart Gür et al. 2009). As suggested in eastern chipmunks (Levesque and Tattersall 2010), lower activity levels due to resting or sleeping in euthermic periods might account for the lower *T*_sub_ in the daily-fed hamsters, although this information is missing in our data set.

An adaptive adjustment of torpor expression in relation to food availability requires the ability to recognize changes in energy availability and to respond adequately. Our specific experimental design ensured that the provision of daily food portions did not result in food shortage but rather represented unpredictable food availability and prevented external energy accumulation. Therefore, the more pronounced use of energy-saving mechanisms in this group would be an adequate response in unpredictable environmental conditions. Hamsters without food stores received enough food as the daily portions were usually not completely consumed. This sufficient energy intake might have allowed them to limit hibernation to a relatively short period and rather use the less energy-saving STBs. An additional benefit might be that changes in the availability of food resources could immediately be detected and allow individuals to quickly adjust their response to improved environmental conditions.

The complete absence of deep torpor in some individuals in our study is in contrast to field data showing that all investigated individuals entered deep torpor (Siutz et al. 2016). The patterns found in the present study resembled those revealed in other studies under semi-natural and laboratory conditions in which food was provided during the winter period outside the hibernacula. These individuals exhibited variation in their temperature patterns and also showed short and/or shallow torpor bouts in addition to deep torpor (Wassmer 2004; Wassmer and Wollnik 1997; Wollnik and Schmidt 1995).

None of the hamsters in our study completely remained euthermic, although sufficient energy reserves would have been available. This supports a bet-hedging strategy, because although large energy reserves are often associated with reduced torpor expression, torpor is usually not completely abandoned (Humphries and Rodgers 2004; Landry-Cuerrier et al. 2008; Levesque and Tattersall 2010; Munro et al. 2005). Among fat-storing hibernators, some individuals even increased torpor expression when in good body condition (Kobbe et al. 2011; Stawski and Geiser 2010; Vuarin et al. 2013). By using torpor, hamsters could not only save energy during winter, but also conserve external energy reserves for being used during the active season. Since under natural conditions, the duration of the winter period as well as food availability and quality after emergence in spring are quite unpredictable, thus remaining food stores could be beneficial for survival and reproduction. An additional advantage would be that animals do not need to leave their burrows, hence minimizing predation risk.

In conclusion, our results demonstrate that food store availability affects torpor expression but particularly highlight the flexible use of torpor and energy-saving strategies in this species, probably reflecting different ways of balancing energy expenditure. While hamsters with unpredictable food availability saved energy primarily by reducing *T*_sub_ and expressing torpor, individuals with access to food reserves seemed to compensate higher energetic demands by consuming their stored food. Both strategies, however, appeared to be successful in terms of body condition as individuals of both groups not only had similar body mass after the experimental period, but also all individuals managed to gain body mass overwinter, which can also occur in free-ranging hamsters (Siutz et al. 2016). The optimal combination of food stores and torpor use could allow individuals to emerge in improved body condition which could positively affect reproductive timing and success in both sexes (Franceschini-Zink and Millesi 2008; Siutz et al., unpublished data). This could be of particular importance in female hamsters as they are capable of producing up to 3 litters per season and previous studies showed a positive relationship between vernal emergence and reproductive timing and success (Franceschini-Zink and Millesi 2008). Field experiments investigating hibernation patterns in response to food supplementation in autumn could give insight into these strategies under natural conditions.
